# Artificial Intelligence Enabled Diagnostics Using Photoplethysmography (PPG): Beyond SpO_2_ and Heart Rate

**DOI:** 10.3390/life16091530

**Published:** 2026-09-15

**Authors:** Divya Dinesh Joshi, Kaaviyashri Saraboji, Asiya Tasleema Shaik, Krishna Sai Kiran Sakalabaktula, Shreya Purohit, Swathi Godugu, Jasmine Nirmal, Riya Kayarkar, Bernardo Henrique Mendes Correa, Namratha Gangidi, Pratibha Yadav, Jyoti Yadav, Farshi Farook, Jieun Lee, Mohammed Naveed Shariff, Divaakar Siva Baala Sundaram, Gayathri Yerrapragada, Poonguzhali Elangovan, Thangeswaran Natarajan, Jayarajasekaran Janarthanan, Sancia Mary Jerold Wilson, Shiva Sankari Karuppiah, Zoma Abbasi, Swetha Rapolu, Simardeep Kaur Bumrah, Rashi Bilgaiyan, Chandra Rupini Premkumar, Anmolpreet Kaur, Lakshmi Sree Pugalenthi, Divyanshi Sood, Suganti Shivaram, Vivek N. Iyer, Vidhya C. M., Simin Masihi, Scott A. Helgeson, Shivaram P. Arunachalam

**Affiliations:** 1 Digital Engineering & Artificial Intelligence Laboratory (DEAL), Mayo Clinic, Jacksonville, FL 32224, USAshreyapurohit321@gmail.com (S.P.); thangeswarann@gmail.com (T.N.); zomaabbasi45@gmail.com (Z.A.); drchandrarupini.pk@gmail.com (C.R.P.);; 2 Department of Medicine, Tulane University School of Medicine, New Orleans, LA 70112, USA; 3 Department of Medicine, St. Vincent Hospital, Worcester, MA 01608, USA; 4 Department of Internal Medicine, UCHealth Parkview Medical Center, Pueblo, CO 81006, USA; 5 Division of Pulmonary Medicine, Department of Medicine, Mayo Clinic, Rochester, MN 55905, USA; 6 Department of Electrical and Computer Engineering, Western Michigan University, Kalamazoo, MI 49008, USA; 7 Division of Pulmonary Medicine, Department of Medicine, Mayo Clinic, Jacksonville, FL 32224, USA; 8 Department of Critical Care Medicine, Mayo Clinic, Jacksonville, FL 32224, USA

**Keywords:** photoplethysmography, artificial intelligence, portal hypertension, pulmonary hypertension, atrial fibrillation, blood pressure, preeclampsia, non-invasive monitoring, compensatory reserve index

## Abstract

Photoplethysmography (PPG) is a non-invasive optical technique commonly used to measure heart rate and oxygen saturation, but its waveform contains additional physiological information that can be analyzed using artificial intelligence (AI). This narrative review summarizes the emerging applications of AI-based PPG in cardiovascular, respiratory, sleep, hemodynamic, pregnancy-related, and portal-hypertension assessment, with the aim of evaluating its potential beyond conventional monitoring and identifying barriers to clinical translation. The literature search was conducted using PubMed, Google Scholar, IEEE Xplore, ScienceDirect, and SpringerLink. Additional relevant studies were identified through screening the reference lists of included articles. Studies published between 2002 and 2026 were identified to capture the development of PPG from conventional monitoring to newer AI-based applications. Human studies were prioritized, while relevant computational, simulated, synthetic, ex vivo, and technical studies were also included. Studies unrelated to PPG, duplicates, and studies with limited relevance were excluded. A total of 96 references were included, covering AI approaches such as convolutional and deep neural networks, ensemble methods, transfer learning, U-Net, generative adversarial networks, and Transformer-based models. Overall, the reviewed evidence suggests that AI-based PPG may support blood pressure estimation, atrial fibrillation detection, sleep and respiratory monitoring, vascular aging assessment, pulmonary hypertension screening, preeclampsia assessment, volume-status and compensatory-reserve assessment, and exploratory assessment related to portal hypertension. However, clinical translation remains limited by motion artifacts, sensor and measurement-site variability, skin-pigmentation-related bias, physiological and environmental influences, heterogeneous methods, limited external validation, and inconsistent clinical and regulatory standards.

## 1. Introduction

Photoplethysmography (PPG), as an optical and non-invasive method for sensing blood volume changes in the microvascular bed of tissue, traditionally employs light transmission and reflection principles [1]. Typically, a PPG waveform is decomposed into two main components: the pulsatile AC wave associated with cardiac activity, and the non-pulsatile DC wave representing impacts from factors like breathing, properties of the tissue bed, thermal regulation, and the autonomic nervous system [1]. Given the simple principle and flexibility, PPG is widely adopted in medical pulse oximeters as well as in common consumer-grade wearables for tracking parameters such as arterial blood oxygen saturation (SpO_2_) and heart rate (HR) [2].

However, the limited application scope for PPG signifies its huge, untapped potential. Beyond monitoring SpO_2_ and HR, PPG waves inherently capture physiological information such as vascular compliance, endothelial function, autonomic tone, and microcirculatory status [3]. However, a large gap between the physiological findings extracted from PPG waveforms and their real clinical applications still exists. This is partly due to the fact that PPG is easily susceptible to noise including movement, background lighting, and physical vibrations. In addition, the morphology of PPG waveforms is sophisticated and affected by multiple physiological factors, thus traditional analytical methods cannot always successfully extract the nonlinear high-dimensional features [4,5].

**Figure 1:**
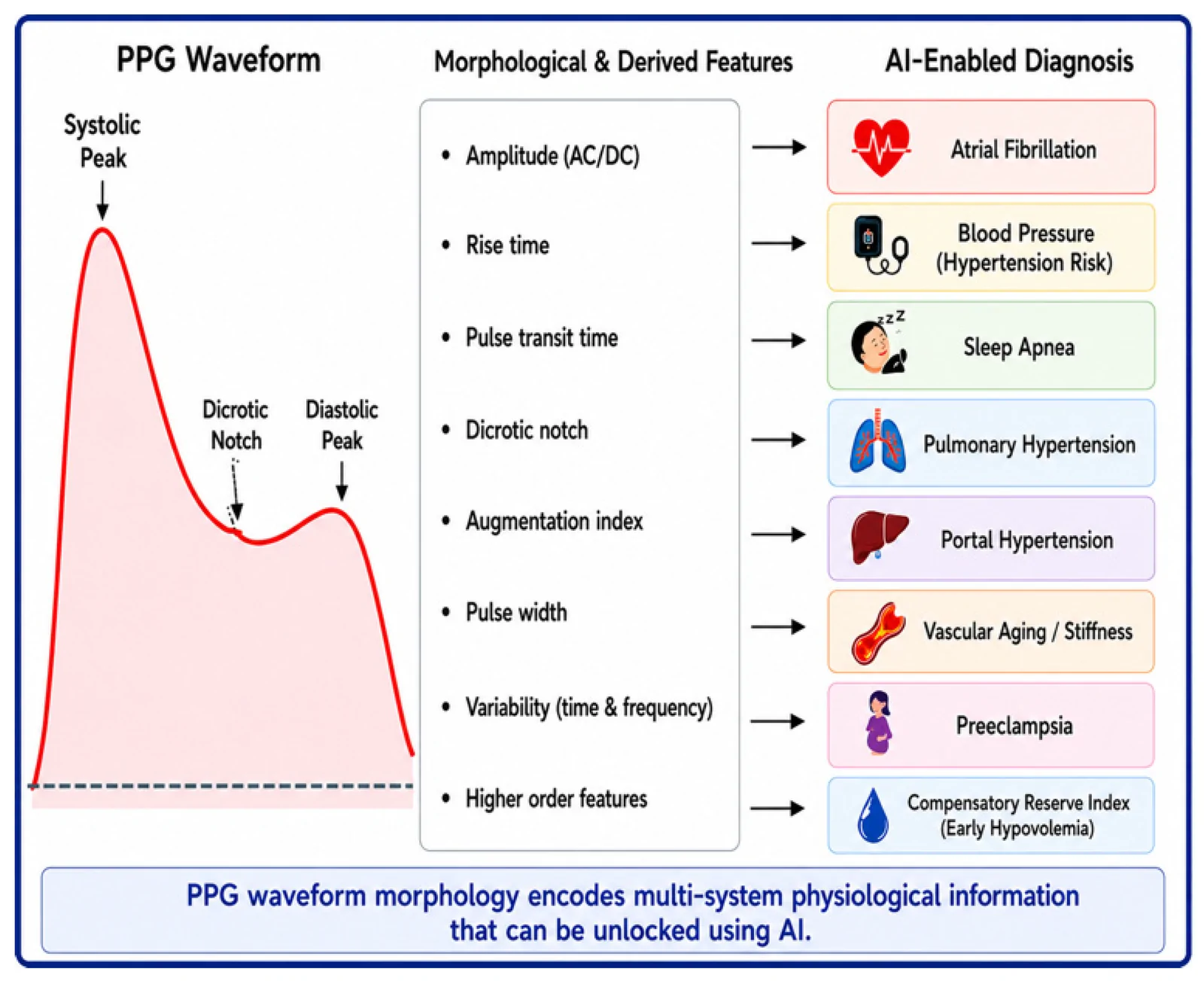
Representative PPG waveform showing the systolic and diastolic components and key morphological features used in PPG analysis.

Traditional analytical methods may not fully characterize all nonlinear, high-dimensional, and subtle patterns inherent in a PPG waveform. As such, the potential of PPG for other diagnostic applications has been underutilized. A typical PPG waveform includes well-known systolic and diastolic components due to cardiovascular physiology, and the main features of a PPG waveform studied in PPG analysis are represented in Figure 1.

Typical PPG interpretation relies on low-dimensional feature extractions such as peak detection or amplitude and frequency analysis, which do not capture all information from within the PPG signals. These methods, although successful for basic monitoring, do not account for minute changes in the waveform indicative of systemic or initial stages of disease states. This limitation has restricted its usage as a diagnostic tool and necessitated more complex procedures, such as catheterization, imaging or laboratory analysis in a wide range of settings. Machine learning (ML) and deep learning (DL), branches of AI (artificial intelligence), are powerful tools capable of processing and analyzing large datasets to remove irrelevant variations and discover underlying patterns [6]. Their application has extended the range of usage into many clinical arenas such as monitoring blood pressure, detecting heart rhythm changes, identifying sleeping apnea, and the diagnosis of pulmonary hypertension, preeclampsia, and aging vasculature [7,8]. Yet most of the relevant reviews concentrate on blood pressure and cardiovascular areas. Other potential PPG applications, such as in pulmonology, obstetrics, or even intensive care medicine, have received comparatively little attention. Furthermore, the literature relating PPG and AI so far tends to focus on a particular disease or application or a specific AI method, rather than offering a broad overview of its implementation into different physiologic systems [9].

Although PPG is mainly used to measure heart rate and oxygen saturation, recent studies show that it may contain other useful physiological information that can be identified using AI. However, these studies are spread across different clinical areas, devices, patient groups, and methods. Therefore, there is a need to bring these findings together to understand the current potential of AI-based PPG and where the evidence is still limited. This review brings together studies from cardiovascular, respiratory, sleep, hemodynamic, pregnancy-related, and emerging portal-hypertension applications and also highlights the main challenges that may limit clinical use. These findings can help identify areas where further research, standardization, and clinical validation are needed. Therefore, this review aims to summarize the current applications of AI-based PPG beyond conventional monitoring and highlight the gaps that need to be addressed before wider clinical use.

### Photoplethysmography Data Acquisition and Analysis

**Figure 2:**
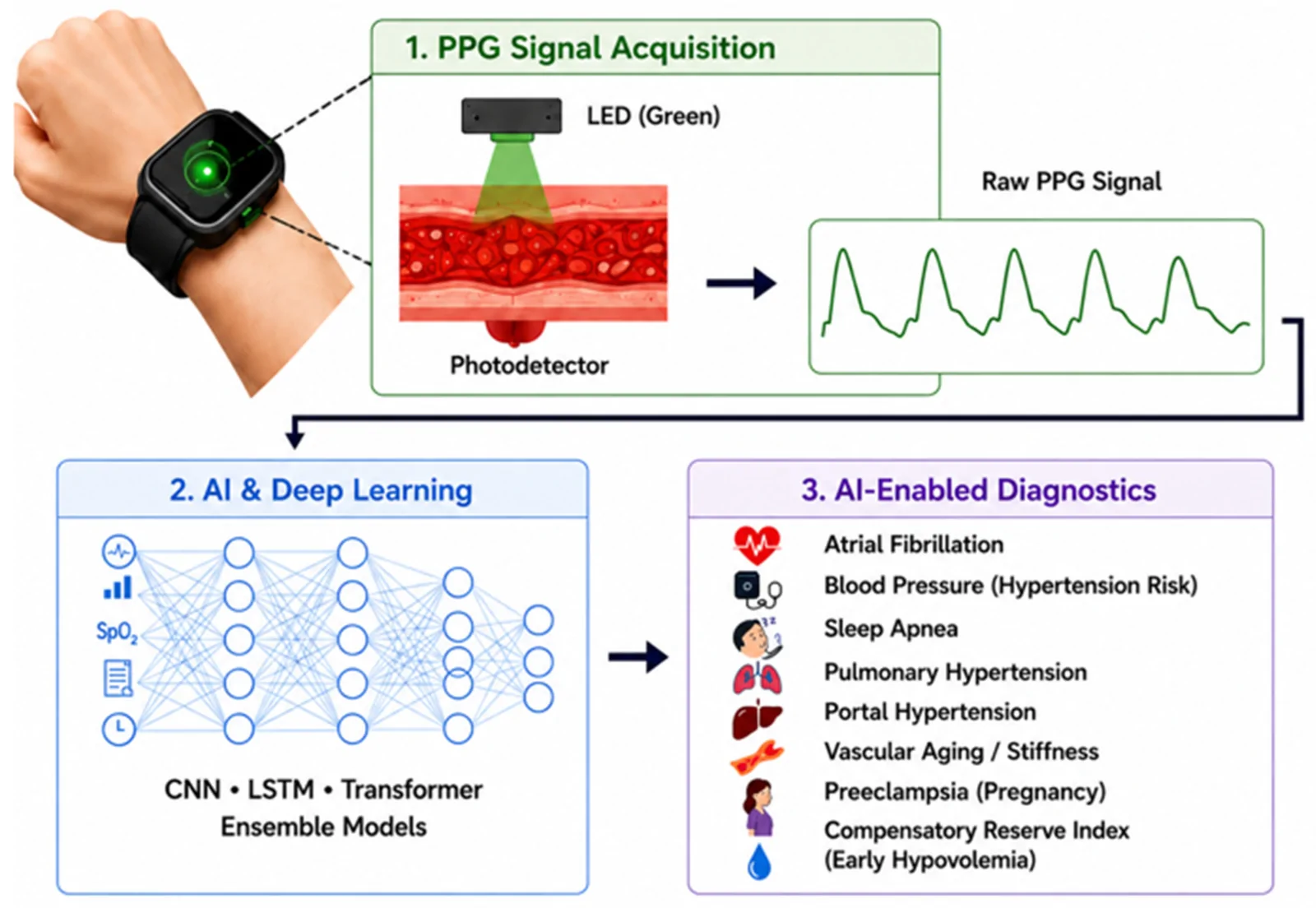
Overview of AI-enabled PPG diagnostics. PPG signals acquired from wearable sensors are processed using artificial intelligence (AI) and deep learning models, including convolutional neural networks (CNNs), long short-term memory (LSTM) networks, and Transformer architectures, to support the detection and monitoring of a wide range of cardiovascular, respiratory, and hemodynamic conditions beyond conventional heart rate and oxygen saturation measurements.

Due to its diverse potential for diagnosis, PPG will go well beyond the basic usages described above and will be a game-changer in many healthcare sectors, providing not only measurements of oxygen saturation and heart rate, but also useful indices related to respiratory function, cardiorespiratory fitness, and neurological conditions, paving the way to a central role in wearable health technology. Indeed, these applications will enhance quality of life by allowing constant individual health-monitoring and will make up the missing part in Public Health, which is real-time distributed measurements. The integration of PPG for medical purposes is not without problems such as motion artifacts, location dependency, and limitations in obtaining readings accurately due to specific clinical contexts, but significant effort have been conducted to address these shortcomings by optimizing the PPG-based device and the processing of the captured PPG signal. Recently, there has been growing interest in extending the application of PPG beyond its more traditional measures to novel and exciting health-monitoring functionalities, as is evident from works such as [10], showing that PPG carries rich and useful diagnostic information beyond the most basic blood oxygen parameters. This information has opened the possibility of a series of relatively simple, inexpensive, and rapid wearable health monitoring technologies with various clinical and fitness uses, as illustrated in Figure 2.

## 2. Role of AI in Interpreting PPG Signals

AI has become an effective tool for PPG signal interpretation. Instead of feature engineering, AI models find the complex nonlinear relationships between the PPG waveforms based on data driven models [11].

Types of AI Models

Previously, classical ML approaches have been used. These methods include SVMs, and random forests [12]. Currently, deep learning approaches like CNNs, LSTMs, and Transformer architectures have been broadly utilized for PPG studies [13]. The former method, CNN, can capture morphological characteristics of the PPG waveforms; meanwhile LSTMs and Transformers are suitable for modeling the long-term temporal dynamics of pulsating signals [14].

### 2.1. Preprocessing of Signals and Noise Reduction Techniques

PPG signal quality degradation is caused by physical activity, ambient light, and biological factors. Some AI pre-processing methods, such as autoencoder-based noise reduction and adaptive filtering, are capable of removing these noises to some degree and preserving useful information for diagnosis [15].

### 2.2. Feature Extraction and Representation

A variety of feature types representing the PPG signal can be extracted through analysis with an artificial intelligence approach, covering a number of feature areas: time domain features, such as variability of inter-beat interval (IBI) and amplitude; frequency domain features, represented by spectrum distribution of power; and morphological features, reflected in waveform shape and inflection points [3,8]. Once the features are available, classification models can determine whether a condition is normal and pathological conditions.

### 2.3. Personalized and Population-Level Modeling

**Figure 3:**
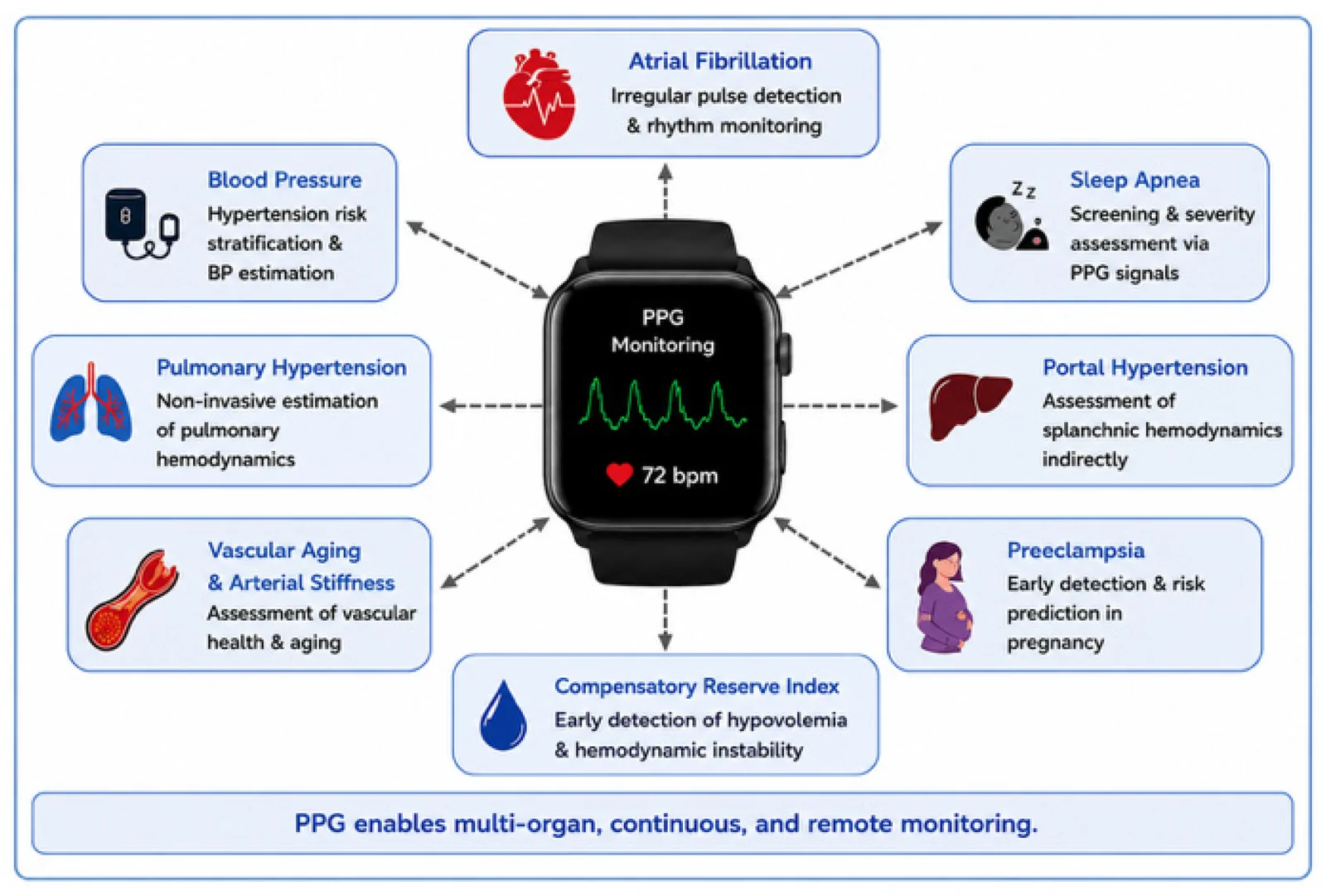
Overview of clinical applications of PPG integrated with artificial intelligence (AI). AI-enhanced analysis of PPG signals enables non-invasive screening, diagnosis, risk stratification, and continuous monitoring across multiple clinical domains, including atrial fibrillation, blood pressure assessment, sleep apnea, pulmonary hypertension, portal hypertension, vascular aging and arterial stiffness, preeclampsia, and compensatory reserve assessment.

Clinicians can now identify diagnostic profiles for an individual patient and a patient cohort by using AI capability to develop population models of normal physiology [16]. Population models support the large-scale implementation of PPG-based screening through wearable devices and telehealth systems. With widely available smart phones, smartwatches, and biosensors, continuous recording and logging physiological signals during daily activities away from the doctor’s clinic have opened up new channels for early disease prevention and management [6,12]. Figure 3 demonstrates an overview of clinical areas where PPG integrated with AI have found emerging applications for diagnosis and monitoring.

### 2.4. Practical Processing of PPG Signals for AI

When analyzing PPG signals using AI methods, the acquired PPG waveform is commonly preprocessed before being input into a machine-learning or deep learning algorithm. PPG signals consist of a pulsatile component dominated by cardiac-induced blood volume changes and a slowly varying baseline component influenced by respiration, vasomotor activity, and various other physiological and measurement-related factors [1,2,3,4]. Therefore, common preprocessing steps may involve filtering, baseline correction, normalization, pulse detection and segmentation, or motion artifact removal techniques [3,4,5]. The resulting signal may be presented to computational analyses as a sequence of individual pulse waveforms, time sequences of fixed duration, derived morphological or temporal features, or others [3,8,9,10].

The required shape of the input data is dependent on the underlying algorithmic approach; classic machine learning approaches rely on manually crafted features extracted from the waveform, whereas deep learning models may learn representations directly from sequences of raw physiological data [6,8,9,10,11,12]. CNNs can identify local patterns in segments of sampled physiological waveforms, while recurrent architecture methods are suited to capturing dependencies over time [11]. Alternatively, Transformer architectures utilize attention mechanisms that allow the modeling of dependencies between sequence elements without recurrent processing [13]. In other approaches, physiological signals may be processed at multiple scales of temporal analysis [12,14]. As such, the input dimension varies widely in PPG-AI work, and is dependent on the sampling rate, analysis window duration, segmentation method, and feature definition used by each study. 

PPG data quality and its completeness is another key aspect to consider when developing real-world AI-based PPG systems. PPG morphology can be affected by both the circumstances of the measurement, such as the measurement method or device setup; the properties of the tissue; and by motion [1,2,3,4,5]. Thus, wearable measurements often include intervals that are noisy or corrupted, as well as missing signal segments, especially during movement. Poor-quality or missing intervals can affect feature extraction and end-to-end inference, and such factors must be taken into account during development and preprocessing. In conclusion, the studies described throughout this review are methodologically heterogeneous, and the reporting of preprocessing protocols, input dimensionalities, and approaches to handling incomplete or poor-quality PPG waveforms varies across studies.

## 3. Blood Pressure Estimation Using PPG Through A.I. Models and Pulse Transit Time Fusion for Clinical Application

Hypertension is the most prevalent chronic condition in the world, affecting many people, and is responsible for approximately 7.5 million deaths worldwide, a large percentage of these being unreported or inadequately treated [17]. It is the primary cause of many fatal diseases including stroke, cardiac diseases and renal failure, so real-time BP measurement is extremely important worldwide [18]. Traditional measurements were performed using a sphygmomanometer with mercury column or oscillometry. However due to the dependence on proper measurement conditions such as cuff placement and prior rest periods for patients before each BP estimation [19], these devices do not lend themselves to continuous monitoring. Due to these limitations, interest in untethered cuffless non-invasive BP monitors has grown considerably.

In this direction, photoplethysmogram (PPG) signals have been extensively studied as it is an optical technique for determining blood volume pulsations in the periphery and has the advantage of being easy to measure optically from any part of the body [20,21]. PPG signals have been well-accepted for applications in heart rate estimation and oximetry. With the advent of ML and AI approaches, a set of feature extraction and recognition methods to extract BP related features from PPG signals have been developed. In the time domain, frequency domain, and morphology domain of PPG signals, time and frequency features, morphological features of the waveform itself, are extracted for systolic BP (SBP) and diastolic BP (DBP) estimation [22]. Moreover, ML/AI models, including DL such as CNNs, RNNs, and Transformers for estimating BP from PPG signals, have been reported [23,24].

For estimating the systolic BP (SBP), the pulse transit time (PTT), defined as the time delay of a pulse wave travelling from the heart to a distal location, has been measured and inversely related to the SBP [21]. Mukkamala et al. developed the signal processing techniques applied to simultaneously recorded PPG and electrocardiogram (ECG) signals to measure PTT, which can measure the beat-to-beat BP. A PPG signal-based continuous BP monitoring framework utilizing various feature sets exhibited an MAE of 9.43 and 6.88 mmHg for SBP and DBP, respectively [22]. A supervised learning model based on CNN was used to estimate SBP and DBP from raw PPG signals with good generalization capability across subjects and signal qualities [23].

To improve BP estimation, other sensor types such as ECG and a combination of multiple sensors have been integrated. ECG and PPG can be used to generate PTT. PTT, measured by the time delay between the R-wave of the ECG signal and the peak of the PPG pulse, showed a strong correlation with BP [24,25]. In comparison to single sensor methods, multimodal signals can complement shortcomings and reduce sensitivity to noise and interruptions [25]. A new and completely novel method was proposed for continuously estimating the BP of a patient from ECG and PPG signals using algorithmic estimation and integrating information such as ECG signal quality scores and continuously iterative BP re-calibrating [24]. A lightweight multimodal learning model was proposed, combining extracted features by CNN and multimodal inputs to effectively address challenges of motion and enhance the robustness of the system compared to a PPG-only approach [25].

While these have all demonstrated significant progress, significant challenges exist in clinical practice for the wide-scale adoption of cuffless, PPG-based BP measurement methods. The variability in skin tones, susceptibility to motion artifacts, electromagnetic interference (EMI) caused by close contact between sensor elements and power sources, as well as the inherent challenges in extracting useful BP indicators from a population with varied health states, will all reduce the effectiveness of such devices [26]. Furthermore, the need to perform routine calibration with traditional cuff-based BP measurement has become a critical stumbling block to achieving “truly” untethered BP monitoring, thereby impacting regulatory acceptance of such devices due to a lack of validation under international standards such as the AAMI and BHS [26]. Table 1 includes selected representative studies of AI-based cuffless BP estimation techniques using PPG and multimodal signals. It shows the typical sensors used, AI-based models applied, performance achieved, and ongoing limitations of each method.

The clinical validation approaches, reported performance, and practical limitations of representative AI-enhanced PPG-based blood pressure monitoring systems are summarized in Table 2.

## 4. Atrial Fibrillation Detection Using AI-Enabled PPG

The most prevalent sustained arrhythmia, atrial fibrillation (AFib), has additional risks of developing heart failure, stroke, and premature death from any cause. Early detection is particularly beneficial in asymptomatic individuals. PPG, a non-invasive optical technique for measuring heart rate and oxygen saturation, is one such area where the advent of artificial intelligence (AI) has led to its widespread availability and consumer deployment, primarily as a tool to determine rhythm [29]. Due to recent advances where AI has become adept at harnessing minor, nuanced characteristics of the PPG waveform to identify AFib with high specificity, the technology is a far more appealing candidate for the consumer and ambulatory space [29].

### 4.1. Morphological and Rhythm-Based Detection via PPG

The PPG data signal obtained from wearable device sensors records pulse waveform features corresponding to cardiac cycles. For AFib, those ventricular events show up as irregular interbeat intervals and variations in pulse waveform shape. Those features, including variability of the interbeat interval, time-domain entropy, and the absence of the dicrotic notch, are common for its identification. Compared to ECG, PPG has the advantage of ease of application and real-time capability but suffers from the inability to measure atrial electrical activity directly. Since P waves are absent in PPG, AFib and some other cardiac arrhythmias like atrial flutter and intermittent PACs can lead to false predictions [30]. Moreover, there are also other challenging factors that can compromise signal quality, such as complexion, lighting environment, and body movements, so efficient preprocessing and artifact detection must be performed by AI.

### 4.2. Contactless PPG Methods

New technologies use standard cameras found on laptops and smartphones to record the PPG signals non-invasively. They work by detecting the subtle fluctuations in skin color that occur with pulsing blood volumes, known as remote PPG (rPPG). The computer system examines specific regions of the face over time while correcting for motion and lighting shifts to calculate a heart rate. Other outside of visual light approaches include using near-infrared imaging, Doppler radar, and time-of-flight sensors to increase the reliability and accuracy of contactless AFib detection. At this time, these techniques are mostly experimental and must be validated in diverse populations and the clinic [31].

### 4.3. Deep Learning vs. Classical Classifiers

Conventional machine learning vs. deep learning on PPG signal-based AFib detection. Various classic ML and deep learning models have been widely used for the task of PPG-based AFib detection. Handcrafted time-domain and frequency-domain features, such as heart rate variability (HRV), pulse interval variability, and various entropy measures, usually serve as the input for classic ML algorithms such as support vector machines (SVMs), random forests, and logistic regression. On the other hand, deep learning methods, including convolutional neural networks (CNNs), recurrent neural networks (RNNs), long short-term memory (LSTM), and Transformer models, can directly learn relevant and informative features from PPG signals. 

For example, CNNs are excellent for characterizing the shape of the waveform, and RNNs/LSTMs excel at capturing time-series dependencies and abnormalities in the rhythm. SQUWA uses attention to minimize the contribution of noisy parts of the PPG signal, thereby enabling robust performance [32]. Table 3 outlines some of the main distinctions between traditional machine learning and deep learning-based methods for AFib classification from PPG signals.

CNN, LSTM, and Transformer models learn different types of information from PPG signals. CNNs mainly learn local patterns in the waveform, while LSTMs learn how the signal changes over time. Transformers use attention to identify relationships between different parts of the signal. Combining these approaches may help capture both local and long-term features. However, model performance depends on the dataset, preprocessing methods, and clinical task. Therefore, results from different studies are difficult to compare, and no single model can currently be considered the best for all PPG applications [13,14].

### 4.4. Continuous Monitoring via Wearable Devices

Most of the major brands (e.g., Apple Watch, Fitbit, Huawei Watch, and Samsung Galaxy Watch) are used to perform passive rhythm monitoring using PPG sensor technology. These devices use AI to alert users about irregular rhythms suggestive of AFib and then request patients take a single-lead ECG reading, when feasible, as confirmation. The Apple Watch 4 and higher provide an FDA-approved ECG feature after an irregular heart rhythm notification [33]. There are many other examples of FDA-approved technology, such as the AliveCor KardiaMobile, which takes a single-lead ECG on demand. Devices that fall between passive screening and full diagnosis use continuous PPG along with periodic ECG recordings to close this gap, increase sensitivity, and reduce the likelihood of false positives [34].

### 4.5. Differentiating AFib from Noise and Other Arrhythmias

It is very difficult to achieve the PPG-based detection of AFib, largely due to the presence of artifacts and mimic rhythms. For example, the irregular nature of motion artifacts can be perceived as a change in the underlying rhythms, while the appearance of a number of PACs or atrial flutter may misidentify it as AFib. Such shortcomings are compensated by several techniques, such as the use of signal quality indices, adaptive filtering, wavelet transform, and ensemble decision rule in advanced deep learning techniques [35]. Deep learning on rich data in adverse conditions also hold promise to discriminate AFib from motion and other abnormal patterns in data. A small number of hybrid devices with a human diagnosis following initial rule-based AI filtering or human verification is used to manage a balance between automated and specific detection [36].

### 4.6. FDA-Cleared Technologies and Ongoing Research

FDA-approved technologies using PPG to detect atrial fibrillation (AFib) reflect an increasing interest in the clinical use of wearable cardiovascular health monitoring devices. Among these are the Apple Watch (Series 4 and later), which provides AFib notifications by analyzing PPG signals, supported by a confirmatory ECG application, and Fitbit wearable devices with an irregular heart rhythm notification feature that leverages PPG [21,22]. Additionally, large population screening studies have successfully illustrated the feasibility of wearable PPG-based detection of AFib. For example, the Apple Heart Study [23], a prospective observational trial involving more than 400,000 participants published in 2019, demonstrated that 84% of individuals receiving irregular rhythm notifications were subsequently diagnosed with AFib through ECG-based testing. Similarly, findings from the Huawei Heart Study also supported the scalability of wearable PPG devices for population-wide screening [24]. Future applications of wearable technology in AFib will incorporate the synergy of various sensor inputs (accelerometers, ECG, and temperature monitors), as well as improved personalization and interpretability of AI models to enhance clinical integration. However, challenges still persist concerning false alarm rates, the avoidance of data bias, and appropriate clinical workflows [37].

Both the Apple Heart Study and Huawei Heart Study used wrist-worn PPG to detect atrial fibrillation (AF), but their monitoring and algorithm methods were different. The Apple Heart Study used a proprietary algorithm that looked for repeated irregular pulse patterns and required multiple irregular readings before sending an alert, helping to reduce false-positive notifications. The Huawei Heart Study used more frequent 60-s PPG recordings every 10 min, which allowed for more chances in which to detect intermittent AF. Huawei reported a PPV of 91.6% compared with 84% in the Apple Heart Study. However, these values cannot be directly compared because the studies differed in sampling frequency, study populations, devices, algorithms, and methods used to confirm AF. Also, neither study reported directly comparable sensitivity and specificity, so their overall diagnostic accuracy cannot be compared reliably [24,37]. Table 4 summarizes the key differences and trade-offs between traditional machine learning and deep learning approaches for PPG-based AFib detection.

## 5. Multimodal AI Framework for Home-Based Sleep Apnea Detection Using PPG-Derived Respiratory Signals, Actigraphy, and Sleep Stage Estimation

### 5.1. Introduction Clinical Need and Rationale for Multimodal Home-Based OSA Detection

Sleep apnea, especially obstructive sleep apnea (OSA), is common and associated with a high burden of cardiovascular morbidity, metabolic disturbances, neurocognitive dysfunction, and impaired quality of life [38]. The diagnosis remains dependent on polysomnography (PSG), which is labor intensive, costly, and challenging to use for large-scale and long-term OSA screening [38]. Thus, wearable, non-invasive alternatives are receiving increasing attention for the screening and identification of OSA.

Recent progress in artificial intelligence (AI)-based diagnosis of sleep apnea has attracted considerable clinical interest, with recently published systematic reviews addressing the potential use of AI in clinical decision pathways for OSA screening and monitoring [39]. As an objective, non-invasive, low-cost device, actigraphy, used to record and estimate sleep–wake cycle patterns, remains a complementary option to PPG signals by providing behavioral context and improving detection performance [40]. The clinical feasibility of actigraphy for sleep monitoring has been demonstrated for many years [41], while advanced actigraphy algorithms are achieving remarkable performance in OSA detection, reaching an epoch-by-epoch sensitivity of 89% [42]. Inspired by these efforts, this review discusses a multimodal AI framework using signals from PPG and actigraphy combined with predicted sleep stages for reliable home-based OSA detection.

### 5.2. PPG-Derived Respiratory Waveforms and Variability

Since multiple physiological phenomena related to respiration modify features of the PPG signal (intensity modulation, baseline wander, pulse-interval variability), PPG is a promising non-invasive modality for monitoring breathing. Reviews and integrative studies have addressed various ways of extracting respiratory signal and rate from PPG, exploring different approaches for estimating the respiratory signal from either intensity-based (IC), frequency-based, or beat-to-beat-based derivations. These studies suggest that the robust extraction of the respiration signal from the PPG is achievable in various physiological locations (finger and forehead) as long as the proper preprocessing of signals is applied [43]. Techniques for instant respiration rate estimation from the PPG of pulse oximeters have also been evaluated on several databases and varied respiratory patterns [44]. Furthermore, a fusion of different PPG modes leads to more accuracy and resistance to noise and motion artifact [45]. Spectral post-processing techniques such as adaptive windowing and spectral smoothing are some of the techniques that have been developed to further increase the reliability of respiratory signal feature estimation [46].

#### 5.2.1. AI (Convolutional Neural Network) Analysis of Nocturnal Pulse Patterns and Desaturation Events

Previous surveys indicate that CNNs and hybrid CNN–temporal networks are among the top-performing algorithms for learning discriminative nocturnal pulse and desaturation patterns from noisy, real-world wearable data [47]. Unlike standard machine learning methods that rely on carefully engineered features, CNNs are able to learn relevant representations directly from raw or partially processed PPG signals. This enables them to identify subtle pulse waveform changes and oxygen desaturation events, which are typical markers of obstructive sleep apnea (OSA) during sleep. CNN-temporal architectures combine local pulse features with temporal variations and have yielded promising results under real-world wearable conditions; however, further validation is required across diverse patient populations and real-world settings.

#### 5.2.2. Integration with Actigraphy and Sleep-Stage Estimation

Complementary information about behavior, such as validated behavioral context supplied by actigraphy, might also be incorporated to support PPG in determining sleep or wake time in the context of ambulatory monitoring [48,49]. This context would be valuable in home OSA assessment, where respiratory abnormalities derived from physiological signals may be interpreted differently depending on whether the person is asleep or awake. Meanwhile, deep learning can also perform sleep-stage estimation using continuous PPG.

SleepPPG-Net illustrated that sleep stages can be estimated directly from continuous PPG using fewer physiological signals than those included in PSG [50].

Integrated respiratory information extracted from PPG, together with movement information collected by actigraphy and information on sleep stages, may provide valuable inputs to a potential artificial-intelligence tool for the home screening of OSA. In a potential approach, PPG provides respiratory-related patterns and pulse dynamics, actigraphy provides movement and sleep–wake context, and the estimation of sleep stages could aid in specifying periods in which respiratory irregularities occur. However, any errors in estimating the sleep–wake state or the stages of sleep itself could contribute to incorrect estimates of the probability of OSA. Such an approach would need to be validated against the gold-standard polysomnography before it could be considered as an alternative to conventional diagnostic testing [49,51].

#### 5.2.3. Performance Against Polysomnography

Accurate classification of sleep stages and breathing events is important for using PPG-based AI in clinical practice. Polysomnography (PSG) is the standard test for diagnosing obstructive sleep apnea (OSA) and is used as the reference test for other methods [51]. PPG-based AI models have been developed to classify sleep stages and detect breathing events using PSG results for comparison. Studies such as Sleep-Net have tested these models on large datasets and compared their results with PSG-based sleep staging. Overall, these studies show that PPG-based AI can provide sleep-related information similar to PSG, although performance can differ between datasets and sleep stages.

#### 5.2.4. Prospects in Home-Based Sleep Diagnostics

PPG respiratory extraction’s robustness and high performance of the developed DL models coupled with well-established actigraphy protocol and a reasonable PSG concordance are providing a more solid foundation for feasible and scalable home sleep diagnosis. Clinical guidelines regarding ambulatory monitoring confirm the value of actigraphy as an addition with reduced physiological signals for obtaining usable laboratory values out of the hospital environment. Key works related to PPG-based respiratory monitoring, artificial intelligence, actigraphy, and home-based sleep diagnosis systems are described in Table 5 along with the key conclusions and restrictions of the studies.

## 6. Pulmonary Hypertension Assessment via AI-Enabled PPG

Pulmonary hypertension (PH) is a complicated and progressive condition that arises as a result of high pressure in the lung’s arteries, thus causing increased blood resistance and eventual right heart failure and premature death. Conventional screening gold standards for PH—such as right heart catheterization and echocardiography—are not only invasive and need specialist expertise, but can also cause diagnostic delays and monitoring problems. The use of non-invasive AI-enabled PPG technology may offer a convenient approach for PH screening [53]. Recently published studies have shown the reliability of PPG using AI-assisted diagnosis for PH. One recent study developed a machine learning classifier that used PPG and OVG (orthogonal voltage gradient) signals to analyze 3000+ physiological features with just brief recordings and achieved a 0.93 AUC with 87% sensitivity and 83% specificity, irrespective of patient age, gender, or PH type. Although these results are promising, they should be interpreted with caution. The model was tested using out-of-fold predictions within the same study dataset and was not validated in an independent external population. In addition, the available dataset was limited for more complex modeling. Therefore, the reported AUC, sensitivity, and specificity show potential, but further testing in different patient populations is needed to confirm that the model can perform reliably in other clinical settings. These findings show that non-invasive screening and the identification of pulmonary hypertension can be achieved by in-depth multidimensional waveform analysis using cutting-edge machine learning techniques.

Another similar approach used waveforms directly with invasive parameters, where pilot studies by Scholte et al. (2024) demonstrated significant correlation (r = 0.68, *p* < 0.001) of the AUC of the waveform with invasively monitored PCWP (pulmonary capillary wedge pressure), demonstrating that peripheral pulse waveforms can interpret intracardiac pressures [54,55]. However, this finding should be considered preliminary because the study was a small pilot investigation conducted exclusively in patients undergoing TAVR for severe aortic stenosis. Although PPG-derived features could distinguish patients with lower and higher PCWP, accurate estimation of the absolute PCWP value remained challenging. Manual selection of high-quality PPG segments may also have introduced selection bias. Thus, the study supports a potential physiological association between PPG features and intracardiac pressure but does not yet establish reliable non-invasive pressure estimation or applicability to broader PH populations.

Broader reviews provide supportive but heterogeneous evidence for the use of AI in PH assessment, where a system-based review of various modalities like imaging and ECG by Attaripour Esfahani et al. (2025) [53] noted the increased use of AI in all of these approaches for the diagnosis and classification of PH, with particular emphasis on the emergence of techniques. A recent meta-analysis by Fadilah et al. (2024) [55] focusing on machine learning approaches in the non-invasive screening of PH reported an aggregated sensitivity and specificity of 85% and 80%, respectively, and was considered as one of the most promising modalities for point-of-care applications when coupled with other data such as demographics and biosignals. However, these results should be interpreted with caution because the studies differed in methods and patient groups, with possible selection bias and limited external validation. Few studies directly compared ML with right heart catheterization. Therefore, the results may not apply to all patients or to PPG-based models [53,55].

Although such developments offer a promising outlook, there is still an urgent need for the improved clinical translation of AI-based models. Current research largely involves small sample sizes and does not incorporate independent multi-center external validation. Signal quality is a major challenge due to a wide range of artifact sources such as peripheral vasoconstriction, skin pigmentation, poor placement, and patient motion. Further prospective studies, standardized validation frameworks, multicomponent integration, and evaluation in real clinical settings are necessary to ensure the reliable widespread deployment of AI-based tools for PH detection and risk stratification [54,55]. This section will also attempt to provide an overview of the state of the current evidence by summarizing all published studies using AI for PH detection either in isolation or together with OVG biosignals. This will encompass all relevant published and in-progress investigational studies, focusing on input signals, feature extraction methods, AI models, diagnostic performance, population samples, and overall study limitations, as illustrated in Table 6.

## 7. Arterial Stiffness and Vascular Aging

PPG is an essential, non-invasive optical technique that has garnered widespread attention in cardiovascular research and clinical practice for its simplicity, accuracy, and ease of use. PPG measures the fluctuations in volume in the peripheral blood vessels by using the variations in the near-infrared light absorbed during the blood pulse to monitor cardiovascular health. The rapid rise in its use is attributed to ease of acquisition, which generally involves small sensors placed at peripheral sites like a fingertip, wrist, or ear [59].

Assessing vascular aging is of great importance as arterial stiffness has proven to be a powerful predictor of cardiovascular morbidity and mortality. As the individual gets older, structural alterations are occurring at the level of the arterial wall (e.g., degradation of elastin and increase in collagen accumulation), which lead to increased arterial stiffness and a reduction in vessel elasticity, increasing the risk of stroke, myocardial infarction, hypertension, and other cardiovascular conditions [60]. Therefore, it is crucial that there exists a low-cost, easy and accurate method of measuring the arterial stiffness of a patient.

Traditional measures for assessing arterial stiffness, such as carotid-femoral pulse wave velocity (cfPWV), are reliable but possess certain practical drawbacks. In addition to being able to access this information only using equipment with extensive training and a multitude of measurement points at varying anatomical locations (which may cause some level of patient discomfort), cfPWV is not practical for screening larger groups of the population or repeated measurements. However, PPG, being a single-site measurement, may overcome some of these limitations, cause less discomfort, and improve patient compliance. However, its use as a substitute for established measures such as cfPWV has not yet been fully established [61].

The utility of AI-based systems has been demonstrated by Shin et al. [59], whose group designed a CNN-based method that is able to precisely predict vascular age by looking at individual pulse signals segment, achieving a mean absolute error of 8.1 years, with a correlation coefficient of 0.61 and an R^2^ of 0.37. They also applied methods such as Grad-CAM to better explain the inner mechanisms of their deep-learning models for a better understanding of what features of the wave they were using. The model, however, could not fully explain the differences in age, and the study population was recruited from a single medical center and consisted of patients undergoing elective surgery, with several clinical conditions excluded. Therefore, the results may not apply to the general population [59].

Furthermore, Chiarelli et al. [60] were also successful in utilizing the benefits of multi-site measurement combined with ECG analysis using deep convolutional neural networks (DCNNs) and predicting the age with remarkably high precision compared to other feature-based techniques, suggesting that machine learning may be useful for extracting age-related information from PPG waveforms. However, the study only included 25 healthy male participants, which limits the generalizability of the findings to women and patients with cardiovascular or other chronic diseases. 

A similarly promising approach has been observed in the analysis of imaging data, as automatic measurements of aortic distensibility from cardiovascular magnetic resonance imaging (MRI) has been used to successfully predict cardiovascular events using a UK Biobank study [62]. However, MRI, despite the accuracy of the measurement of aortic distensibility, can be problematic due to requirements for substantial investment in infrastructure, resources, and expertise. Hence, a PPG-based method is a more affordable and accessible solution compared to MRI-based measurements.

The potential of PPG is augmented by its use in non-invasive measurement through readily available devices. The ubiquity of smartphones with built-in sensors has already introduced a way of continuously monitoring cardiovascular health in the everyday lives of millions of people [59]. This could potentially support population-level screening of vascular aging, although differences in smartphone hardware, signal quality, and measurement conditions may affect the consistency of PPG recordings [61]. The potential of machine learning for estimating aortic PWV from PPG-derived pulse transit time has also been demonstrated by Huttunen et al. using simulated cardiovascular data [63]. However, because the study was based on virtual subjects rather than real patients, its findings may not directly translate to real-world clinical populations [63]. These findings highlight the potential applications of this technology in population screening, patient monitoring, and remote care. However, larger clinical studies, external validation, and standardized measurement protocols are needed before AI-based PPG can be widely adopted for vascular-age assessment and cardiovascular risk monitoring [61]. 

In essence, it offers a confluence of clinical accuracy and practical affordability for a broad range of vascular age applications. The growing adoption of AI/ML tools, coupled with the ubiquitous availability of sensing hardware, positions it as a uniquely valuable asset for preventive cardiology. In conclusion, PPG combined with AI/ML shows promise as an accessible approach for vascular-age assessment and cardiovascular monitoring. However, differences in signal acquisition, study populations, and validation methods remain important challenges. Further clinical validation and standardization are needed to determine whether these approaches can be reliably implemented in routine practice.

## 8. Pre-Eclampsia Prediction via AI-Enhanced Photoplethysmography

Preeclampsia is a significant cause of maternal and perinatal morbidity and mortality worldwide, with an onset and progression that are often difficult to predict. This causes problems in diagnosing the disease early. Traditional clinical risk factors, as well as blood pressure measurements, are insufficient predictors of the disease. Recently, there has been increasing interest in where vascular tone, arterial stiffness, and autonomic nervous system function can be captured, even through subtle physiological changes characteristic of the pathology of pre-eclampsia. When combined with AI, it has demonstrated the ability to improve the early diagnosis of pre-eclampsia. 

For instance, Euliano et al. (2018) analyzed a number of features from signals, including heart rate variability and pulse wave analysis (PWA), in pregnant women, concluding that more than 80% sensitivity in distinguishing women who would later develop pre-eclampsia from normotensive controls could be achieved through beat-to-beat timing variability and pulse wave indices [64]. However, performance was lower when pre-eclampsia was compared with hypertensive controls, suggesting that PPG features may have limited ability to distinguish pre-eclampsia from other hypertensive conditions [64]. By using more complex features that extract characteristic changes in the shape of waveforms associated with pre-eclampsia, it was identified that a decreased falling scaled slope, which reflects vascular compliance, could serve as a biomarker that signals pre-eclampsia onset several weeks before clinical symptoms occur. Specifically, the values for the falling scaled slope in pregnant women who later developed pre-eclampsia were significantly lower than those in unaffected pregnancies (mean 0.19 compared with 0.24, *p* < 0.001), indicating abnormal vascular function preceding the development of clinical signs of pre-eclampsia (Chen H et al., 2023) [65].

The study conducted by Munyao et al. (2024) [66] in a real-world setting found that a “pre-eclampsia watch” that continuously recorded signals in pregnant women, combined with the assessment of maternal vital signs and AI-based algorithms, achieved an accuracy of 77% in predicting early preeclampsia risk using a Naive Bayes classification model. This provides evidence that a low-cost ambulatory screening tool is feasible for identifying high-risk populations, even in low-resource settings. The Naive Bayes model achieved 77% accuracy, demonstrating feasibility but also indicating that further validation is needed before clinical implementation [66].

Although not PPG-based, the Butler et al. (2024) [67] study demonstrates a significant advance in the biosignal-based prediction of pre-eclampsia. Their models, based on 12-lead ECG signals, identified women at risk of early-onset pre-eclampsia up to 90 days before clinical diagnosis with an AUC of 0.98. This demonstrates that ECG biosignal analysis is a viable approach for risk stratification in pre-eclampsia. Finally, de Kat et al. (2019) [68] studied various AI models for predicting the occurrence of pre-eclampsia and observed that models incorporating biosignals and ECG provided greater accuracy than models based only on clinical data. Nevertheless, the authors emphasize that larger prospective studies and standardized protocols are still required before widespread real-world implementation.

## 9. Portal Hypertension Prediction Using Photoplethysmography

### 9.1. Introduction

Portal hypertension (PHT) is an important and frequently observed complication in chronic liver disease, particularly cirrhosis. PHT is instrumental in the pathogenesis of the main clinical manifestations, including variceal hemorrhage, ascites, hepatorenal syndrome, and hepatic encephalopathy. To assess the degree of portal hypertension, hepatic venous pressure gradient (HVPG) measurement remains the method of choice. Significant threshold values of HVPG > 10 mmHg predict future complications, while values of ≥12 mmHg are associated with an increased risk of variceal rupture. A reduction in HVPG greater than 20% from basal values, or to <12 mmHg, has also been associated with improved outcomes and reduced mortality. However, HVPG measurement is invasive and expensive, and it can only be carried out in referral centers. In recent years, there has been increasing demand for practical and easy-to-use non-invasive methods for detecting and assessing the course of PHT, as well as monitoring treatment response, and this has been identified as an important priority in the international guidelines of the Baveno V Workshop [69].

### 9.2. Pathophysiological Basis of Portal Hypertension

Portal hypertension develops as a consequence of both fixed and dynamic factors in cirrhosis. These factors contribute to elevated intrahepatic vascular resistance (IHVR), resulting in part from structural alterations (e.g., fibrotic matrix and regenerative nodules), and importantly, from increased tonic contraction. Increased tonic contraction is stimulated by the contraction of activated hepatic stellate cells (HSC) and myofibroblasts and exacerbated by impaired endothelial function in the hepatic vasculature. Increased vascular tone is caused, on the molecular level, by the reduced availability of NO and the upregulation of vasoconstrictors such as endothelin-1. In this regard, endothelial eNOS dysfunction due to a reduction in eNOS phosphorylation and accumulation of NOS inhibitors, such as ADMA, is particularly important [69]. The opposite is the case in extrahepatic vessels that produce an increased amount of NO, leading to peripheral vasodilatation and thereby increased splanchnic blood flow, which in turn increases the portal pressure. Furthermore, enhanced signaling by angiogenic factors including VEGF and PDGF promotes angiogenesis, the development of portosystemic collaterals, and contributes to sustaining the hyperdynamic state of circulation [69].

### 9.3. The Need for Non-Invasive Evaluation

With the central role of systemic and splanchnic hemodynamics in the progression of portal hypertension, the interest in the development of non-invasive methods to recognize such alternations has become stronger. Conventional imaging methods like Doppler sonography, CT, and MR may identify portosystemic collaterals and changes of blood flow to some extent but are expensive and require extensive experience in the interpretation of findings. Serum biomarkers such as ET-1 and U-II—the most potent vasoconstrictors—have correlated with HVPG in some studies but are still hampered in their clinical application with variability and without sufficient validation.

Even if many advances have been made in the field of non-invasive portal hypertension evaluation, many of the current techniques face several limitations. Biomarker-based methods, i.e., the ALBI and Lok scoring systems, correlate only moderately with hepatic venous pressure gradient (HVPG) and are not sufficiently specific for a definite diagnosis. CT, MRI, and their radiomic-based derivations, although they improve the accuracy of diagnosis, suffer from being expensive, difficult to access, are accompanied by radiation exposure (CT), and have a time-consuming procedure for post-processing. Elastographic methods including transient elastography (TE), point shear wave elastography (pSWE), and 2-dimensional SWE (2D SWE) have shown moderate to excellent correlations with HVPG, but may have technical failures in the evaluation of patients with obesity or ascites and are highly operator-dependent [70]. Doppler sonography (DUS), despite its widespread availability, has been found to be inconsistent for its correlation with portal pressure depending on the respiratory phase, equipment, and the patient’s condition. On the other hand, contrast-enhanced ultrasound (CEUS), especially microbubble-based techniques for pressure assessment like subharmonic-aided pressure estimation (SHAPE), shows excellent correlation with HVPG and useful hemodynamic information, but its invasiveness (intravenous contrast) and limited availability, occasional failure to obtain results from some patients as well as its technical limitations make it less convenient for wider use [71].

PPG, a common and non-invasive optical measurement tool used in cardiovascular health monitoring, has potential for research in portal hypertension because it can detect real-time peripheral hemodynamic alterations, including changes in vascular tone, pulse transmission characteristics, and waveform morphology. However, these PPG-derived features do not directly measure portal pressure or HVPG; rather, they may serve as indirect physiological proxies of the systemic and peripheral hemodynamic changes associated with portal hypertension. Considering its cost-effectiveness, portability, and compatibility with wearable devices, it may therefore serve as an adjunctive non-invasive tool for evaluating or monitoring patients with portal hypertension, although its clinical validity remains to be established [71].

### 9.4. Photoplethysmography for Volume Status Assessment in Cirrhosis: A Potential Tool for Portal Hypertension Monitoring

Earlier research has shown that PPG waveform analysis during a Valsalva maneuver can strongly correlate with intravascular volume status in patients suffering from systolic heart failure. While the Valsalva maneuver at end-Valsalva is physiologically coupled with a decrease in cardiac filling, this response is blunted during the fluid-overloaded state. Whereas earlier attempts relied on the simplistic ratio of a resting versus during-Valsalva measurement of the PPG signal amplitude to approximate pulse pressure changes, more recent applications of signal processing and machine learning have enabled the extraction of more detailed information about waveform shape to infer more complicated physiological states, including systemic vascular resistance, arterial stiffness, and continuous blood pressure. Similar signals derived from PPG have also been examined for the detection of fluid responsiveness in surgery, mechanical ventilation, and shock states.

Little work, however, has extended the application of PPG waveform analysis to estimate intravascular volume status in patients suffering from cirrhosis, in whom fluid overload commonly coexists with portal hypertension. In a recent prospective study, Mazumder et al. examined the ability of PPG waveform analysis to estimate volume status in cirrhotic patients undergoing non-urgent cardiac catheterization [72]. Following informed consent, patients underwent a standardized seated Valsalva maneuver, and PPG signals were recorded using a finger probe before, during, and after the maneuver. PPG waveforms were subsequently analyzed using machine learning approaches to identify physiologically meaningful features, while pulmonary capillary wedge pressure (PCWP) and/or left ventricular end-diastolic pressure (LVEDP) were measured during cardiac catheterization. Volume overload was defined as PCWP and/or LVEDP greater than 15 mmHg [72]. Among the 39 patients for whom both PPG testing and catheterization data were obtained, features identified through machine learning approaches predicted the presence or absence of volume overload with predictive performance comparable to BNP-based models. Machine learning classifiers trained on PPG features, including support vector machines and logistic regression, were able to discriminate between volume-overloaded and control patients with a high degree of accuracy, substantially outperforming a simple dummy classifier [72].

Therefore, the data presented support the potential feasibility of using simple, bedside-derived PPG waveform features obtained during a Valsalva maneuver to non-invasively detect intravascular volume status in cirrhotic patients, potentially as a proxy marker of underlying hemodynamic changes related to portal hypertension, especially in the context of a fluid-overloaded state. This non-invasive technique needs to be validated in a larger series of cirrhotic patients as a proxy tool to monitor clinical status and response to treatment in patients with portal hypertension.

Importantly, this study did not directly measure portal pressure or HVPG. Rather, PPG-derived features were used to assess intravascular volume status using PCWP and/or LVEDP as reference measures, and therefore represent an indirect physiological proxy rather than a direct measurement of portal hypertension. Larger studies comparing PPG-derived features directly with HVPG are required to establish their validity for portal hypertension assessment.

### 9.5. Photoplethysmographic Amplitude-to-Pulse Pressure Ratio as a Marker of Vascular Compliance in Circulatory Dysregulation

It has been hypothesized that the ratio of the photoplethysmographic amplitude to pulse pressure (PPGamp/PP) can serve as a dynamic surrogate for vascular compliance during acute circulatory changes. In a prospective observational study involving patients undergoing orthotopic liver transplant, PPG and invasive arterial pressures were recorded simultaneously on a beat-to-beat basis during graft reperfusion, a condition associated with rapid vasodilation and circulatory failure. After reperfusion, there was a substantial increase in the PPGamp/PP (median [IQR]: +41.9 [16.7–95.9%]) and Windkessel-derived arterial compliance (C_wk, +41.7 [15.0–77.9%]), as well as a marked decrease in total peripheral resistance (TPR, −45.7 [−55.8 to −24.1%]). 

PPGamp/PP demonstrated a strong inverse correlation with TPR (median r = −0.80) and a strong positive correlation with Cwk (median r = 0.86), suggesting that PPG_amp/PP could be used to dynamically measure the response of vascular tone and compliance to vasodilatory signals [72]. While this study was limited to liver transplant recipients, the pathophysiological consequences that resulted—specifically reduced vascular resistance and increased vascular compliance—are similar to those present in the hyperdynamic circulatory condition associated with portal hypertension. 

Given its relatively simple, noninvasive nature and susceptibility to changes in peripheral vasodilation, the amp/PP ratio may prove useful as an indirect physiological marker of vascular alterations in patients with cirrhosis and portal hypertension. However, PPG-based assessment of portal hypertension remains investigational and has not been validated as a direct measure of portal pressure or HVPG.

Table 7 provides an overview of the current diagnostic and monitoring methods for portal hypertension compared with PPG, highlighting the relative benefits, drawbacks, and clinical validation status of each approach.

### 9.6. Imaging Photoplethysmography (iPPG): Real-Time Perfusion Mapping

Splanchnic and hepatic hemodynamic alterations associated with portal hypertension are complex, but existing techniques for quantifying these hemodynamic changes are invasive, limited, or expensive. In a recent proof-of-concept study, Kamshilin et al. (2022) [73] demonstrated the potential of imaging photoplethysmography (iPPG), an ECG-synchronized, contactless, dye-free optical imaging technique, to perform non-invasive, real-time intraoperative quantitative tissue perfusion assessment during stomach and colon surgery. Imaging photoplethysmography was applied in 14 surgical procedures to demonstrate changes in tissue perfusion before and after anastomosis, and the perfusion findings were consistent with the surgeons’ intraoperative assessments. Spatial perfusion maps were reconstructed in tissues affected by vascular ligation, devascularization, and vascular reconstruction despite challenges such as tissue motion, tissue-type diversity, and variations in blood vessel lumen size. The study further demonstrated, for the first time, the feasibility of using iPPG to generate perfusion maps of intra-abdominal organs and to detect subtle real-time perfusion changes using only optical light signals. These preliminary findings suggest that iPPG may have potential applications in portal hypertension research, where real-time, non-invasive assessment of abdominal organ perfusion remains an unmet clinical need [73].

### 9.7. Potential Role and Future Directions for PPG-Based Hemodynamic Monitoring

Cumulatively, this points to a valid case for the evaluation of PPG in monitoring volume status, vascular compliance, and mapping intra-abdominal perfusion. The indirect nature of the current evidence does not detract from the clear physiological link—portal hypertension leads to systemic vasodilation, increased cardiac output, and peripheral alterations in circulation that may be evident in the PPG waveform signature. Future work needs to be undertaken to see whether PPG indices, in isolation, or with further machine learning support, and importantly, under controlled maneuvers (such as Valsalva maneuvers), could serve as non-invasive biomarkers in monitoring disease progression or therapeutic response in portal hypertension.

## 10. PPG Waveforms, CRI, and Non-Invasive Measures of Hemodynamic Stability

### 10.1. Introduction

The compensatory reserve index (CRI) is a new parameter that describes a person’s physiological reserve prior to hemodynamic decompensation. Machine learning algorithms that analyze peripheral arterial waveforms have been used to provide CRI-related information for point-of-care assessment of cardiovascular status [73]. The CRI ranges from 1 to 0, representing physiological normovolemia with maximum compensatory capacity and the onset of hemodynamic decompensation, respectively, in the setting of hypovolemia [64]. Estimation of the original CRI was derived from volume-clamping methods for continuous blood pressure (BP) measurement, which are not ideal for point-of-care settings because of their large size and high cost [74,75]. Other newer modalities such as PPG, tonometry, and ultrasound arrays address this limitation by monitoring arterial waveforms analogous to BP waveforms [74]. Data obtained from a PPG device, such as a pulse oximeter, can be interpreted to assess an individual’s CRI in any clinical setting for the early identification of impending hemodynamic decompensation [75].

### 10.2. CRI Reflects Compensatory Mechanisms During Hypovolemia Using PPG Waveform Features

In early stages of acute hypovolemia, multiple compensations are invoked by the ANS, and the result is seen in heart rate, stroke volume, cardiac output, blood pressure, and peripheral vascular resistance, all in the attempt to maintain blood flow to peripheral tissues. As hypovolemia worsens, these compensations fail, and patients enter decompensated shock. Changes in the classic vital signs of heart rate and blood pressure are often very minimal in the early compensation stages of hypovolemia, making them a poor indicator of pending decompensation shock. CRI, on the other hand, has been proven to be significantly more sensitive, specific, and accurate in detecting decompensated shock than any standard vital signs [76,77].

The Compensatory Reserve Measurement (CRM), developed at the U.S. Army Institute of Surgical Research, was conceived as a triage tool for determining the degree of physiological compensation in patients with hypovolemia. Initially, Latimer et al. [78] defined the Compensatory Reserve Measurement (CRM) as:

CRM = 1 − (BLV/BLVHDD)

where BLV = actual blood loss volume and BLVHDD = the volume of blood loss required to result in hemodynamic decompensation. As these actual blood volumes cannot readily be assessed in a clinical situation, various computational algorithms were developed for estimating the CRM based on salient features identified within a patient’s arterial waveform. An arterial waveform is composed of an “ejected wave”, which reflects the compensations affecting cardiac function, and a “reflected wave”, modulated by the responses regulating the periphery. The changes in the shape and features of these waveforms vary in response to circulatory volume and the attendant compensation mechanisms inherent to each patient.

An approach to simulate central hypovolemia in a clinical setting by applying lower body negative pressure (LBNP) and acquiring arterial waveform data in human volunteers until hemodynamic decompensation has allowed the calculation of a CRM based on LBNP exposure as a surrogate for blood loss:

CRM = 1 − (LBNP/LBNPHDD)

where LBNP = current negative pressure level applied to the volunteer, and LBNPHDD = the LBNP level at which the volunteer reaches hemodynamic decompensation. The system utilizes machine learning to extract features of ejected and reflected waves from PPG waveforms that are correlated with each level of LBNP exposure, from the baseline (no pressure; CRM = 1) to the point of hemodynamic decompensation (CRM = 0). Machine learning allows for the identification of how each of these features maps to an LBNP level between 1 and 0, such that the system can be trained to provide a continuous estimation of physiological reserve. Ultimately, the goal is for the system to be capable of calculating a subject’s physiological reserve using solely waveform data, without prior knowledge of their baseline or demographic information [79].

### 10.3. CRI in Hemorrhage and Trauma (LBNP Models and Clinical Studies)

CRI is an accepted measure in trauma and hemorrhage patients to reflect physiological reserve and indicate the onset of decompensated shock. Latimer et al. [78] conducted a recent study demonstrating that lower prehospital CRI trend values are associated with a greater need for early blood transfusion. This demonstrates the ability of CRI to identify hemorrhagic shock before traditional clinical signs become evident. Although not as prognostic as systolic blood pressure and the shock index, CRI provides portability and has the added benefit of assessing physiological reserve, making it appropriate for use in the prehospital environment or in resource-limited settings. In another study conducted by Johnson et al. [80] involving a cohort of 89 trauma patients, CRI was a significantly more sensitive predictor of the need for urgent interventions, such as blood transfusion or surgery, than systolic blood pressure. CRI demonstrated superior sensitivity and negative predictive value. This demonstrates that the ability of CRI to predict early physiological deterioration makes it a useful triage tool in emergency settings.

### 10.4. Use in Dengue Shock and Appendicitis to Detect Severity and Monitor Response

CRI has also been validated in a variety of infectious diseases, including Dengue Shock Syndrome (DSS). In a prospective, observational ICU study involving 63 patients with DSS, CRI performed well in predicting re-shock within 12 h (AUC = 0.84), significantly outperforming heart rate and pulse pressure. A cutoff value of less than 0.4 yielded the best balance between sensitivity and specificity (66% and 86%, respectively), and CRI values declined approximately two hours before the onset of re-shock, providing an early warning for clinical intervention [81]. While little evidence is available for conditions such as appendicitis, it is plausible that CRI could be valuable in the setting of septic or preoperative hypovolemia, where substantial cardiovascular compensatory responses often mask changes in other vital signs.

### 10.5. Correlation with Gold Standards: Stroke Volume, Vascular Compliance, and Total Peripheral Resistance 

The study by Convertino et al. [74] found relationships between CRI and conventional hemodynamic variables during LBNP. Heart rate increased during the LBNP protocol, demonstrating an inverse relationship with CRI; however, it did not serve as an early indicator of decompensation. Systolic and diastolic blood pressure remained stable up to −45 mmHg of LBNP in both low- and high-tolerance subjects, making these measurements poor predictors of early hypovolemia. SpO_2_, respiratory rate, and end-tidal CO_2_ did not change throughout the protocol and therefore were not reliable predictors of central hypovolemia. Stroke volume declined linearly with increasing LBNP in both the high- and low-tolerance groups. Although the rate of decline was similar, the high-tolerance group exhibited a greater absolute reduction in stroke volume before the onset of decompensation, suggesting that stroke volume can predict hypovolemia to some extent. CRI decreased markedly during LBNP, with the low-tolerance group reaching CRI values of 0.6 and 0.3 significantly faster than the high-tolerance group, indicating reduced compensatory reserve. While CRI does not directly measure vascular compliance, it reflects waveform features influenced by arterial compliance and therefore provides an indirect estimate of changes in vascular compliance. Total peripheral resistance is expected to increase during cardiovascular compensation; however, CRI is more sensitive for detecting the earliest physiological changes.

### 10.6. Potential for Real-Time CRI Monitoring via AI-Enhanced Wearable Pulse Oximeters 

CRI technology can also be incorporated into AI-based, wearable monitors that collect PPG waveforms from the fingers, wrist, or forehead using reflective or transmissive optics, where AI algorithms can be used to interpret these complex waveforms in real-time. An AI-driven Compensatory Reserve Measurement (CRM) algorithm, which forms the basis of two FDA-approved medical devices, analyzes subtle changes in the PPG signal over time to determine an individual’s compensatory reserve [76]. Convertino et al. [74] trained and evaluated three AI models—logistic regression, decision trees, and deep learning—using LBNP data to estimate CRM. All three models performed well in estimating CRM, with the deep learning model demonstrating the highest accuracy, even when waveform segments were shortened to just 10 s, without requiring the patient’s demographic information. Because these AI-based algorithms do not require patient demographic information and provide personalized, real-time estimates of CRM, they may have significant utility in prehospital and military settings for continuous monitoring of cardiovascular stability, the early identification of potential decompensation, and timely intervention. 

In summary, significant variations were found between the extent of the evidence across AI-PPG applications. To this point, there appears to be an increasing body of evidence on AFib screening and blood pressure assessment compared to the other PPG-related conditions with more research involving wearables and larger sample sizes, including multiple algorithms, both machine and deep learning-based, for evaluating both conditions. More recently, there has been encouraging work focusing on the application of AI-PPG detection for sleep apnea and vascular aging; however, the studies often differ in methodology for acquiring PPG signals as well as in the reference standard applied and populations utilized, making definitive comparison challenging. Encouraging progress has also been made for pulmonary hypertension and pre-eclampsia, though less data exist, with a limited number having had independent external validation. Portal hypertension has a more preliminary evidence-base, with the research available demonstrating improvements in associated hemodynamic parameters, such as volume status, arterial compliance, and tissue perfusion, but not a direct measurement of portal pressures or HVPG. Taken together, these finding indicates that AI-PPG-related conditions are at varying degrees of clinical maturity.

## 11. Challenges and Future Directions for PPG in Medicine

### 11.1. Introduction

In less than two decades, PPG has evolved from bedside pulse oximeters to a format that has seen its integration into wristbands, phones, and camera-based monitors. Its rise is attributable to its low cost, wearable comfort, and the wealth of information contained within a waveform that provides data on pulse rate, rhythm, vascular timing, and indirect measures of autonomic activity. However, the promise of this technology has not yet matched dependable clinical performance, and for PPG to transition into mainstream medicine, several hurdles must be overcome, including challenges related to motion interference, skin/perfusion-induced variability, interpretability, validation standards, hardware optimization, and clinical outcome evidence, all of which continue to limit the full diagnostic potential of PPG.

### 11.2. Technical and Signal-Quality Challenges

#### 11.2.1. Motion Artifact

Even today, motion still constitutes the primary technical obstacle for PPG. Various real-life activities such as walking, gestures, and sweating generate motion artifacts that can potentially hide the cardiac pulsatile signal. While traditional methods like accelerometer-based subtraction can remove many of these artifacts, they become less effective when the spectral ranges of the motion and pulse frequencies overlap [82].

Several approaches, including multichannel sensors, Independent Component Analysis (ICA), and Empirical Mode Decomposition (EMD), have successfully mitigated artifacts in controlled studies. More recently, machine learning-based algorithms, such as lightweight edge models and generative adversarial networks (GANs), have shown improved performance in artifact detection and signal reconstruction [82,83,84].

A study conducted by Yu L, Li H, Zhang T, et al. on Tiny-PPG, a lightweight deep neural network optimized for edge-device applications, demonstrated an artifact detection accuracy of 87.4% [85]. Traditional algorithms based on signal quality indices (SQIs) and time- and frequency-domain morphological features have achieved an accuracy and specificity of approximately 90%, while significantly reducing heart rate (HR) error under resting conditions [86]. Nevertheless, algorithms developed using restricted datasets with limited testing often fail to generalize well to real-world clinical populations and complex motion patterns. Therefore, prospective clinical validation remains uncommon.

#### 11.2.2. Site, Contact Pressure, Pigmentation, and Perfusion

There is significant variability in signal amplitude and fidelity between anatomical sites, tissue thickness, local perfusion, and skin pigmentation. Differences in the location of measurement and the posture of the measurement subject can also alter blood flow and blood pressure because of hydrostatic changes in position relative to the heart. In addition, differences in tissue thickness and structural properties affect PPG morphology, as the transmission or reflectance of light through full-thickness skin depends on the layered tissue architecture, where the avascular epidermis overlies the vascular dermis.

Transmittance PPG measured at the fingertips therefore produces waveforms with higher fidelity than reflective PPG measured at the wrist, which is more susceptible to motion and changes in perfusion [87].

Changes in the pressure applied between the device and the skin can also lead to significant PPG waveform distortions, including changes in signal amplitude, particularly in the pulsatile AC component, because of partial arterial occlusion and the resulting changes in local blood flow. In Ref. [88], systolic, mean, and diastolic blood pressure were estimated using PPG signals while external pressure was applied to an artery.

Importantly, optical devices based on PPG principles, including pulse oximeters, have demonstrated measurable bias in individuals with higher skin pigmentation, leading to the underestimation of hypoxia and potentially influencing downstream clinical decision-making [87]. It is therefore essential to conduct studies using diverse cohorts and to report performance stratified by skin tone, age, and perfusion state.

#### 11.2.3. Physiological Confounders and Interpretability

In addition to motion artifacts and skin pigmentation, several other physical factors can alter PPG signals and complicate their clinical interpretation. Changes in vascular compliance, tone, and local perfusion arising from age, temperature, sympathetic activity, vasoactive drug use, or sudden changes in volume status can alter pulse amplitude and shape, sometimes mimicking pathological conditions [89]. The site of measurement, together with tissue thickness, also influences the optical path length and scattering, causing signal variability between different measurement sites, even within the same individual [88]. Although machine learning classifiers can recognize complex waveform patterns associated with these factors, purely data-driven “black-box” models do not reveal the physiological processes underlying their predictions. Therefore, high-stakes clinical applications should incorporate mechanistic priors, such as explicit models of vascular mechanics and optical physics, into the learning framework to ensure that performance is maintained in the presence of these confounding factors while also allowing clinicians to understand why a particular result was generated.

### 11.3. Data Standards and Validation

Comparisons among existing PPG studies have consistently highlighted inconsistencies in reference standards (ECG versus invasive arterial lines versus third-party oximeters), variability in motion protocols, and non-standardized error reporting across studies. Recent consensus and benchmark papers have recommended standardized workflows with pre-registered protocols, together with shared, task-specific error metrics for cuffless blood pressure (BP) estimation, including reporting mean error and standard deviation alongside trend- and trajectory-based metrics, and validation against appropriate gold standards under both resting and motion conditions [83]. Communications Medicine recommends a standard evaluation framework for PPG-based BP estimation, while *Scientific Data* provides a community benchmark and curated datasets designed to support fair cross-model comparisons [84]. Newly published peer-reviewed datasets now combine synchronized PPG with ECG and either invasive or oscillometric BP measurements, and *PLOS Digital Health* extends this benchmarking approach beyond BP estimation to include PPG-ECG for heart rate variability, PPG-Doppler for stroke volume, and multi-site PPG for pulse wave velocity. Several of these open datasets also incorporate controlled variables such as contact pressure, temperature, posture, ambient light, and motion, enabling reproducible and direct comparisons of model performance while facilitating accurate performance evaluation and the identification of remaining challenges [44,86]. Toolkits are most useful when they are developed using such diverse, clinically relevant datasets and report their results using agreed-upon evaluation metrics.

### 11.4. Clinical and Regulatory Integration

Although most wearables utilizing PPG-enabled technology are marketed as consumer products, few have received regulatory clearance to monitor critical vital signs like heart rate and oxygen saturation. Any clinically used devices require clearly defined failure modes, transparent data governance, and ease of integration into electronic health records. Both over-alerting leading to false alarm fatigue and missed critical events resulting in compromised patient safety require pre-specified algorithms, prospective validation in target populations, and well-defined use cases for regulatory clearance [88,90]. Contactless photoplethysmography (remote-contact photoplethysmography, rPPG) presents the promise of scalable, infection-conscious monitoring, but systematic reviews consistently call for rigorous validation against invasive or otherwise accepted clinical gold standards before it is adopted at the bedside [91].

### 11.5. Emerging and Potential Clinical Applications

#### 11.5.1. Pulmonary Hypertension (PH)

The PPG waveform consists of timing indices and morphological features that reflect central hemodynamics. Initial proof-of-concept studies have combined PPG with optical or impedance signals for the noninvasive screening and monitoring of pulmonary hypertension in situations where invasive assessment is not practical. However, these methods remain preliminary and require validation in larger prospective cohort studies using right-heart catheterization as the reference.

#### 11.5.2. Pregnancy and Pre-Eclampsia

In pregnancy, subtle changes in vascular compliance and autonomic tone precede the clinical onset of pre-eclampsia. Several cohort studies using PPG-derived pulse-wave metrics and heart-rate variability have demonstrated promising potential for the early detection of gestational hypertension and risk stratification for pre-eclampsia [92]. Better integration with existing uterine and fetal monitoring systems, together with validation in large and diverse obstetric populations, is needed before these approaches can be introduced into clinical practice.

#### 11.5.3. Diabetes and Glucose Monitoring

Continuous glucose monitoring remains invasive and burdensome because current systems require a sensor that penetrates the skin. Using signal segmentation together with deep learning approaches such as ResNet and TinyML, real-time noninvasive glucose estimation has achieved clinically acceptable accuracy, with a root mean square error of approximately 19.7 mg/dL and 100% of measurements falling within Zone A of the Clarke Error Grid [92,93]. Furthermore, diabetes detection using signals and deep learning models (e.g., CNN-GRU) has demonstrated strong performance and good generalizability across multiple datasets. However, these findings are based on early proof-of-concept studies, and the clinical feasibility of noninvasive glucose monitoring remains uncertain due to challenges in reproducibility, independent validation, and agreement with the established clinical standards. Therefore, PPG-based glucose monitoring should currently be considered an experimental approach rather than a clinically established application.

#### 11.5.4. Hormonal Biomarkers

Wearable optical sensing is also being explored for endocrine biomarker monitoring. By combining multi-wavelength PPG (MW-PPG) with feature extraction methods such as independent component analysis (ICA) and heart-rate variability (HRV) analysis, researchers are investigating noninvasive testosterone level prediction using deep learning regression models [93]. However, this work remains highly experimental and is based on preliminary proof-of-concept research. Larger studies with independent clinical validation are required before the feasibility and clinical usefulness of PPG-based hormonal biomarker monitoring can be established.

**Figure 4:**
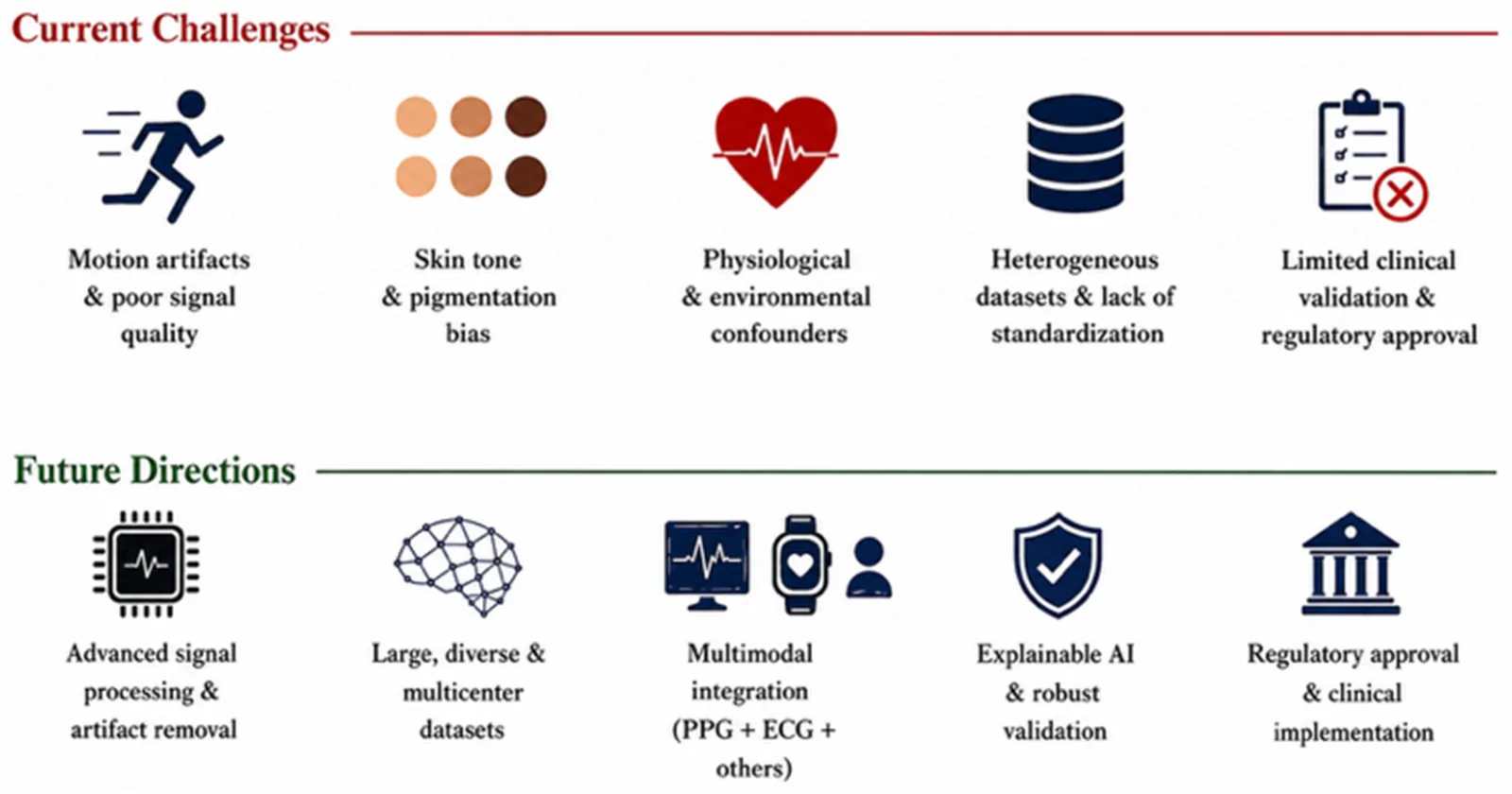
Challenges, Limitations, and Future Directions.

In addition to the promising applications highlighted above, several technical, methodological, and clinical challenges continue to limit the widespread adoption of AI-enabled PPG technology. At the same time, advances in multimodal biosignal integration, explainable artificial intelligence (XAI), wearable sensor technology, and personalized predictive modeling are creating new opportunities for clinical implementation. Figure 4 summarizes the major challenges, current limitations, and future research directions for the continued development of AI-enabled PPG technologies.

### 11.6. Standardization Challenges

A major challenge in the clinical use of AI-driven PPG systems is the lack of standardization across the measurement process. Differences in sensor hardware, optical configuration, signal acquisition, preprocessing, feature extraction, datasets, and validation methods can affect the recorded signal, and in turn, the performance of AI models. Therefore, differences in reported model performance may not always be due to better algorithms but may also result from differences in how the signal was collected and processed. Recent work has highlighted the need for greater standardization in four main areas: hardware and reporting, signal acquisition, signal processing and analysis, and validation for clinical use. Future studies should use clear reporting standards for sensor specifications and acquisition methods, provide transparent preprocessing methods, use diverse datasets that include different devices and populations, and perform external validation across devices and populations. Collaboration between researchers, device manufacturers, clinicians, and regulatory bodies will be important for developing common standards while still allowing innovation in technology [94,95].

## 12. Future Directions

PPG is a promising source of physiological information, but its use in clinical practice is still at an early stage. Further research is needed to improve sensors, signal processing, AI methods, and clinical validation. Combining PPG with ECG, seismocardiography, and motion sensing may help reduce motion-related errors and improve signal quality, but these methods need further testing [95]. Explainable and physiology-based AI may also help make PPG results easier to understand and more reliable [96]. Future studies should use larger and more diverse patient groups and assess clinical outcomes. This is important to determine whether PPG-based applications, such as atrial fibrillation screening, pre-eclampsia screening, and pulmonary hypertension monitoring, provide real clinical benefit and are cost-effective. 

Recent studies have explored several ways to extend PPG-based blood-pressure assessment. Wearable devices may support ambulatory cardiovascular monitoring [97], and deep-learning methods have been used to reconstruct arterial blood-pressure waveforms in critically ill patients [97,98]. Blood-pressure variability is also being studied as a potential digital biomarker [99]. Other recent work includes interpretable PPG-based models for classifying blood-pressure stages [99] and preliminary evaluations of cuffless continuous monitoring in intensive-care and after cardiac surgery [100,101]. Future studies should prioritize diverse, multi-ethnic training and validation datasets to improve the generalizability of AI-based rPPG models. Dasari et al. found no significant differences in pulse-rate estimation between Indian and Sierra Leonean participants or between genders, although errors increased with subject movement and changing illumination. These findings suggest that demographic differences do not necessarily result in measurable bias, but also highlight the importance of evaluating rPPG models across diverse populations and real-world conditions. Further validation across populations with varied skin tones, body composition, vascular characteristics, and environmental conditions will be important to establish robust and generalizable performance.

Although these findings are encouraging, larger multicenter studies involving diverse patient populations are needed to assess accuracy, generalizability, calibration requirements, and clinical usefulness before these approaches can be adopted routinely [98,99,100,101,102,103].

## 13. Conclusions

Artificial intelligence-enhanced PPG offers a non-invasive approach with the potential to be applied beyond basic pulse oximetry to hypertension, atrial fibrillation, pulmonary and portal hypertension, pre-eclampsia, sleep apnea, arterial stiffness, vascular aging, diabetes, and hemodynamic instability through the Compensatory Reserve Index (CRI). Together with sophisticated analytics, PPG could enable the early detection of disease, improved risk stratification, and continuous patient monitoring, all contributing to precision and preventive healthcare. While significant advances have been made, AI-enhanced PPG is still under investigation, and its clinical translation is limited by motion artifacts, variability in skin pigmentation and perfusion, physiological confounds, heterogeneous reference standards, limited datasets for developing the underlying AI models, and the lack of robust validation frameworks and outcome-driven clinical trials. Future research needs to be driven by multidisciplinary teams focusing on the development of multimodal sensors, physiology-informed and explainable AI models, and large, annotated datasets for standardization and reproducibility. Robust multicenter clinical trials are needed to determine whether PPG-based interventions, including screening for atrial fibrillation, prediction of pre-eclampsia, monitoring of pulmonary and portal hypertension, assessment of vascular aging, detection of sleep apnea, and CRI-based monitoring, improve patient outcomes and are cost-effective. Overall, AI-driven PPG is an exciting area of research that, once the current technical and translational gaps are addressed, may contribute to the development of precision and preventive healthcare.

## Figures and Tables

**Table 1:** AI-Based Approaches for Cuffless Blood Pressure Estimation Using PPG and Multimodal Signal Fusion.

Study (Year)	Paper Type/Data	Key Findings/Performance	Limitations
Samimi & Dajani, 2023 [26]	Calibration-free cuffless BP; PPG/cardiovascular dynamics	Demonstrated calibration-free BP estimation; BHS Grade A and AAMI-related validation reported	Limited BP-related information in PPG; interpretability, robustness, and physiological validation remain a concern
Arjomand et al., 2025 [23]	Transformer; PPG-only	Transformer-based BP estimation from a single PPG signal	Further external and clinical validation required
Mukkamala et al., 2015 [16]	ECG–PPG; PTT-based analysis	PTT, measured from the ECG and PPG signals, was used for beat-to-beat BP estimation	Requires dual-sensor measurements; calibration-related limitations
Kachuee et al., 2017 [17]	PPG signal-based continuous BP monitoring; feature-based ML	MAE of 9.43 mmHg for SBP and 6.88 mmHg for DBP	Requires further validation for broader clinical/wearable applications
Slapničar et al., 2019 [9]	CNN using raw PPG signal	Demonstrated BP estimation with good generalization across subjects and different signal qualities	Generalization to real-world wearable conditions remains a challenge
Liu et al., 2020 [27]	ECG–PPG features; ML models; invasive arterial BP reference	Random forest achieved the best performance; RMSE was 5.87 ± 3.13 mmHg for SBP and 3.52 ± 1.38 mmHg for DBP	Small cohort of 35 clinically stable patients with arrhythmias; no independent external validation and limited generalizability
Mahmud et al., 2022 [21]	PPG + ECG; Shallow U-Net	AI-based BP prediction using multimodal PPG and ECG signals	Requires multimodal sensing; external validation remains important
Yousefian et al., 2020 [22]	BCG + PPG; PTT–PWA fusion	Wearable cuffless BP trend tracking using multimodal physiological signals	Requires multiple sensors/signals; focused on BP trend tracking rather than fully validated absolute BP measurement

AI models that do not require calibration have the potential to enable scalable PPG-based BP monitoring. Recent advances in deep learning have demonstrated the feasibility of estimating arterial BP waveforms directly from PPG signals without cuff-based calibration. However, fundamental challenges remain in accurately mapping PPG characteristics to arterial pressure, highlighting the need for improved interpretability, robust signal processing, and rigorous physiological validation of AI-based BP estimation models. Beyond algorithmic performance, several clinical and practical challenges may hinder the adoption of AI-based PPG BP monitoring in routine clinical practice, including inconsistent ambulatory performance, sensitivity to motion artifacts, calibration requirements, limited external validation, and lack of interoperability across wearable devices.

**Table 2:** Clinical Validation, Performance, and Limitations of AI-Enhanced Blood Pressure Monitoring Systems.

Study (Year)	Method/Model	Key Findings	Limitations
WHO, 2025 [28]	Global epidemiological data	1.28B affected; high burden in No tech/device evaluation	Epidemiological data; no specific device evaluation
Forouzanfar et al., 2017 [15]	Epidemiology GBD study	Hypertension/elevated BP represents a major global cardiovascular health burden.	Population-level, no evaluation of cuffless BP technology
Stergiou et al., 2018 [19]	BP device validation protocols	Highlights the importance of standardized validation protocols for BP-measuring devices.	Existing validation protocols may not fully address emerging cuffless technologies
Avolio et al., 2022 [20]	Cuffless BP clinical challenges	Identifies calibration, validation, motion artifacts, skin tone, and regulatory issues affecting cuffless BP adoption.	Lack of standardization and adequate validation limits clinical adoption

**Table 3:** Traditional Classifiers vs. Deep Learning for AFib Detection Using PPG [31].

Criteria	Deep Learning Models	Classical Classifiers
Algorithms	CNN, RNN, LSTM, Transformers	SVM, Random Forest, Logistic Regression
Feature Engineering	Not required (end-to end learning)	Required (HRV, entropy, etc.)
Input Type	Raw waveform	Handcrafted features
Sensitivity/Specificity	High (AUC > 0.95 in many studies)	Moderate to high (AUC 0.80–0.94)
Robustness to Noise	High (especially with attention mechanisms)	Lower (sensitive to motion artifacts)
Interpretability	Low (black-box models)	Higher (e.g., decision trees offer explainable logic)
Resource Requirements	High (training + inference)	Low to Moderate
Deployment	Challenging without optimization	Easier on low-power or embedded devices

**Table 4:** Comparison of Traditional Machine Learning and Deep Learning Approaches for AFib Detection Using PPG Signals.

Type	Algorithms	Input	Perf.	Pros	Cons
Classical ML	SVM, Random Forest, Logistic Regression	Handcrafted features (HRV, RR interval SD, entropy) [32]	Moderate to high	Interpretable, low resource, easy deployment	Needs feature engineering, noise sensitive [32,33,34,35]
Deep Learning	CNN, RNN, LSTM, Transformers (with attention e.g., SQUWA)	Raw PPG waveform	High; AUC > 0.95 reported in many studies [32]	End to end learning, captures waveform, helps reduce noise	Black-box, high compute, harder deployment

**Table 5:** Summary of PPG, AI, Actigraphy, and Home Sleep Studies.

Study (Year)	Method	Findings/Insights	Limitations
Charlton et al., 2017 [44]	Systematic review of ECG/signal processing	Respiratory waveform recovery feasible with proper processing	No new data; performance depends on sensor placement Conceptual
Meredith et al., 2012 [45]	Review of physiological basis and processing	PPG-derived respiratory rate feasible across datasets	Different derivation methods and respiratory patterns may affect performance [45]
Karlen et al., 2013 [46]	Smart Fusion of respiratory-induced frequency, intensity, and amplitude variation extracted from PPG	Combining the three estimates showed a trend toward lower respiratory rate estimation error than individual methods	Evaluated in 29 children and 13 adults; estimates affected by artifacts or disagreement between methods were rejected
Nilsson, 2013 [47]	Review of respiratory modulation in PPG signals	Respiratory modulation of the PPG waveform can support respiratory rate monitoring	Patient movement and movement at the probe-tissue interface can interfere with the signal
Jiang et al., 2023 [39]	1D-CNN on raw	>90% accuracy for sleep apnea detection	Further validation across diverse populations and real-world settings is required [39]
Choksatchawathi et al., 2024 [52]	Deep learning using features extracted from fingertip PPG signals	Improved sensitivity, specificity, and AUROC relative to the comparison models on two benchmark datasets	Evaluation used existing sleep-laboratory datasets; prospective validation in routine clinical settings remains needed
Sadeh & Acebo, 2002 [42]	Clinical review of actigraphy for sleep–wake assessment	Actigraphy helps document sleep-wake patterns and responses to behavioral and medical interventions	Less reliable during prolonged motionless wakefulness or altered movement patterns; accuracy depends on the device, scoring algorithm, and population
Morgenthaler et al., 2007 [48]	AASM practice parameters based on systematically graded evidence	Supports actigraphy for assessing sleep patterns, circadian rhythms, and treatment response in selected populations; may estimate total sleep time in OSA when PSG is unavailable	Recommendations depend on the population and clinical purpose; further research is needed to broaden clinical applications
Kotzen et al., 2023 [40]	SleepPPG-Net: deep learning for four-class sleep staging from continuous raw PPG	Median Cohen’s κ of 0.75 on the held-out test set; κ of 0.74 on an external database after transfer learning	External database performance was evaluated after model adaptation; this does not establish equivalent performance without adaptation
Gil et al., 2010 [49]	PPG pulse-rate variability in children	PRV correlates with sleep-disordered breathing	Pediatric population; adult applicability is uncertain
Kapur et al., 2017 [51]	AASM clinical practice guideline for adult OSA diagnostic testing	Supports technically adequate home sleep apnea testing in uncomplicated adults at increased risk of moderate-to-severe OSA	PSG is recommended after a negative, inconclusive, or technically inadequate home test, and for patients with specified complicating conditions

**Table 6:** Artificial Intelligence Models for Pulmonary Hypertension Detection Using Photoplethysmography and Multimodal Biosignal Analysis.

Study (Reference)	Signal Inputs	Feature Engineering/Preprocessing	AI/ML Model Type	Performance (AUC, Sensitivity, Specificity, etc.)	Dataset/Cohort	Key Notes
Zhang & Ma (2023)—Software Eng. & Applications (China) [56]	PPG only (fingertip)	Preprocessing with wavelet scattering; features learned via pre-trained CNN (GoogLeNet) on PPG waveform	CNN with fine-tuning	Accuracy ~97.8%; Sens ~96%; Spec ~98%; F1-scores ~0.96	*N* = 216 PPG recordings with invasive PAP labels (RHC as gold standard)	Pilot single-center study; validated on hold-out test split; potential overfitting; no external validation yet
Nemati et al. (2024) —Diagnostics (MDPI)—Analytics4Life/CorVista [57]	PPG + OVG (orthogonal voltage gradient)	Hand-crafted library of ~3298 features capturing electro-mechanical synchrony; reduced to 216 features	Elastic Net logistic regression + Random Forest stacking (ensemble)	AUROC ≈ 0.93; Sens 87%; Spec 83%; consistent across subgroups	Multi-center development dataset; symptomatic patients with RHC and healthy volunteers	Developed point-of-care algorithm; robust; validated internally; awaiting independent external validation (achieved later)
McLean et al. (2025) [58]	PPG + OVG (multimodal cardiac signals)	Proprietary machine-learned algorithm combining electrical and perfusion metrics	Supervised ML classifier (based on prior ensemble approach)	AUROC = 0.95 (mPAP ≥ 25 mmHg); Sens 82%; Spec 92%; at mPAP ≥ 21 mmHg: AUC = 0.93, Sens 78%, Spec 92%; NPV > 99%	*N* = 462 symptomatic patients; multi-center (18 U.S. sites); all RHC confirmed; train/test split	Clinical validation of CorVista PH Add-On; first FDA-cleared PPG + OVG PH test; generalizable across PH etiologies; comparable to echocardiographic screening

**Table 7:** Comparison of Non-Invasive Tools for Assessing Portal Hypertension.

Modality	Advantages	Limitations	Stage of Validation
HVPG (Gold Standard) [69]	Accurate measurement; strong prognostic value	Invasive; costly; requires expertise; limited availability	Fully validated
Transient Elastography (TE) [70]	Quick; bedside; correlates with fibrosis and portal pressure	Limited by ascites, obesity; operator-dependent; not always predictive of complications	Widely used; Baveno endorsed
2D SWE/pSWE [70]	Real-time imaging; correlates with HVPG	Requires technical skill; variability in measurement; limited use in severe cases	Emerging validation
CT/MRI Radiomics [70]	Detailed anatomical and functional info; can assess collaterals and flow	Expensive; radiation (CT); complex processing; limited reproducibility	Moderate validation in research
Contrast Enhanced Ultrasound [70]	Visualizes perfusion and collaterals; SHAPE correlates with HVPG	IV contrast required; limited success rate; equipment and operator-sensitive	Promising; early validation
Serum Biomarkers (e.g., U-II, Endothelin-1) [70]	Non-invasive; accessible via routine blood draws	Poor specificity; influenced by comorbidities; lack of standard cutoffs.	Low; investigational
Photoplethysmography [72,73]	Low-cost; portable; real time vascular data; wearable compatible	Not liver-specific; limited studies of cirrhosis; indirect measure of portal pressure	Experimental; early human data

## Data Availability

This review was based on publicly available academic literature databases.

## Abbreviations

The following abbreviations are used in this manuscript:

PPG	Photoplethysmography
AI	Artificial Intelligence
ML	Machine Learning
DL	Deep Learning
ECG	Electrocardiography
BP	Blood Pressure
AFib	Atrial Fibrillation
OSA	Obstructive Sleep Apnea
PH	Pulmonary Hypertension
HVPG	Hepatic Venous Pressure Gradient
CRI	Compensatory Reserve Index
CNN	Convolutional Neural Network
LSTM	Long Short-Term Memory
PTT	Pulse Transit Time
HRV	Heart Rate Variability
PSG	Polysomnography
PWA	Pulse Wave Analysis
